# Multi-omics analysis of the dynamic role of STAR+ cells in regulating platinum-based chemotherapy responses and tumor microenvironment in serous ovarian carcinoma

**DOI:** 10.3389/fphar.2025.1545762

**Published:** 2025-03-03

**Authors:** Hongwei Lan, Weihua Yan, Xiao Huang, Jiali Cui, Helei Hou

**Affiliations:** ^1^ Precision Medicine Center of Oncology, The Affiliated Hospital of Qingdao University, Qingdao, Shandong, China; ^2^ Department of Pathology, The Affiliated Hospital of Qingdao University, Qingdao, Shandong, China; ^3^ Department of Oncology, The Affiliated Hospital of Qingdao University, Qingdao, Shandong, China

**Keywords:** serous ovarian carcinoma, chemoresistance, cancer-associated fibroblasts, STAR, wnt signaling

## Abstract

**Background:**

Serous ovarian carcinoma (SOC) is the most lethal subtype of ovarian cancer, with chemoresistance to platinum-based chemotherapy remaining a major challenge in improving clinical outcomes. The role of the tumor microenvironment (TME), particularly cancer-associated fibroblasts (CAFs), in modulating chemotherapy responses is not yet fully understood.

**Methods:**

To explore the relationship between CAF subtypes and chemotherapy sensitivity, we employed single-cell RNA sequencing (scRNA-seq), bulk RNA-seq, spatial transcriptomics, immunohistochemistry (IHC), and immunofluorescence (IF). This multi-omics approach enabled the identification, characterization, and functional analysis of CAF subtypes in both chemotherapy-sensitive and chemotherapy-resistant SOC patients.

**Results:**

We identified steroidogenic acute regulatory protein-positive (STAR+) cells as a novel CAF subtype enriched in chemotherapy-sensitive SOC patients. STAR + cells exhibited unique transcriptional profiles and were functionally enriched in pathways related to P450 drug metabolism, lipid metabolism, and amino acid metabolism, with enhanced pathway activity observed in chemotherapy-sensitive groups. Spatial transcriptomics and IF revealed that STAR + cells were closely localized to tumor cells, suggesting potential cell-cell interactions. Further communication analysis indicated that STAR + cells may suppress WNT signaling in tumor cells, contributing to improved chemotherapy responses. Importantly, STAR expression levels, validated by IHC, were positively correlated with chemotherapy sensitivity and improved patient prognosis. Platinum-based chemotherapy was shown to increase the proportion of STAR + cells, underscoring their dynamic response to treatment.

**Conclusion:**

Our study identifies STAR + cells as a novel CAF subtype that enhances chemotherapy sensitivity in SOC. By modulating key metabolic pathways and potentially suppressing WNT signaling, STAR + cells could contribute to improved treatment responses. These findings position STAR + cells as a promising biomarker for predicting chemotherapy efficacy in SOC, which warrants further investigation.

## 1 Introduction

Serous ovarian carcinoma (SOC) is the most lethal form of ovarian cancer, primarily due to its high invasive capacity and resistance to chemotherapy (Liu et al., 2020; Liu et al., 2019). Platinum-based chemotherapy combined with surgery remains the first-line treatment for SOC, achieving an initial response in most patients. However, approximately 80% of patients experience relapse, leading to a 5-year survival rate of less than 30% for stage IIIC-IV patients (Ahn et al., 2024; Gockley et al., 2017; Gadducci and Cosio, 2021; Lheureux et al., 2019). Despite advances in targeted therapies and immunotherapy, their efficacy in SOC remains limited, leaving few effective options for platinum-resistant patients (Konstantinopoulos and Matulonis, 2023; Marchetti et al., 2021). The frequent recurrence of SOC underscores the urgent need to understand and address the mechanisms driving its chemoresistance to platinum-based therapies.

SOC’s genetic landscape is characterized by extensive copy number variations (CNVs) and recurrent mutations in key genes such as TP53 and BRCA1/2, which contribute to its genomic complexity and therapy resistance (Raab et al., 2024; Dias et al., 2021; Montemorano et al., 2024). In addition to genetic factors, the tumor microenvironment (TME), particularly cancer-associated fibroblasts (CAFs), has emerged as a crucial driver of tumor progression and treatment resistance (Carvalho et al., 2022).

Specifically, in tumors such as non-small cell lung cancer and colorectal cancer, CAFs enhance tumor cell resistance to chemotherapy agents like fluorouracil and platinum-based drugs by secreting or receiving exosome signals (Wang et al., 2021; Hu et al., 2015). Targeting CAFs can increase drug absorption by the tumor, thereby improving the efficacy of cancer chemotherapy (Loeffler et al., 2006). CAFs contribute to chemotherapy resistance through mechanisms such as increasing drug efflux, reducing drug absorption, promoting EMT, and creating a supportive microenvironment via extracellular matrix remodeling and immune cell infiltration, allowing tumors to survive under therapeutic pressure (Helms et al., 2020; Zhao et al., 2022). However, recent studies on CAF subtypes have revealed that CAFs exhibit significant heterogeneity, with distinct subtypes potentially playing divergent roles in drug response (Ma et al., 2023; Lavie et al., 2022; Su et al., 2018). Some types of CAFs have tumor-suppressive properties (Feng et al., 2022). For example, CD146+ CAFs have been identified as tumor-suppressive subsets in breast cancer, with high levels of CD146+ CAFs increasing the sensitivity of breast cancer to treatment (Brechbuhl et al., 2017). Similarly, Slit2+ and CD146+ CAFs can suppress tumorigenesis and enhance chemosensitivity (Mhaidly and Mechta-Grigoriou, 2021). However, the specific contributions of these CAFs to platinum resistance in SOC remain largely unknown.

To comprehensively analyze changes in the TME of SOC across patients with varying responses to chemotherapy, we identify and characterize a novel subtype of CAFs-steroidogenic acute regulatory positive (STAR+) cells. By integrating multiple omics analyses of SOC with real-world clinical data, we aim to elucidate the role of STAR + cells and their interactions with tumor cells in mediating responses to platinum-based chemotherapy. Through a detailed characterization of the cellular and microenvironmental dynamics, we seek to provide new insights into the therapeutic vulnerabilities of SOC, paving the way for improved treatment strategies.

## 2 Methods

### 2.1 Transcriptomics data sources

In this study, transcriptomics data were obtained from publicly available datasets. Specifically, single-cell RNA sequencing data (GSE211956, GSE201047, GSE184880) were downloaded from the Gene Expression Omnibus (GEO) repository (https://www.ncbi.nlm.nih.gov/geo/), and rc47y6m9mp.1 was retrieved from Mendeley (https://data.mendeley.com/datasets/rc47y6m9mp/2). Spatial transcriptomics data (GSE211956) were also sourced from the GEO repository. Bulk RNA sequencing data (GSE227100, GSE28739, GSE156699, GSE30161) were downloaded from GEO; TCGA-OV data were retrieved from The Cancer Genome Atlas (TCGA) repository (https://portal.gdc.cancer.gov/); normal ovarian tissue data were downloaded from UCSC (https://xenabrowser.net/datapages/?cohort=GTEX). Prognosis assessment data were accessed from Kaplan-Meier Plotter (https://kmplot.com/), ROC Plotter (https://rocplot.com/), and the TIMER2 database (http://timer.cistrome.org/).

Patients were classified as either platinum-based chemotherapy-sensitive or chemotherapy-resistant based on two criteria: (1) chemotherapy-resistant patients were defined as those with progression-free survival (PFS) ≤ 6 months after the last platinum-based chemotherapy, or a histological assessment showing no or minimal tumor response (CRS = 1); (2) chemotherapy-sensitive patients were defined as those with PFS >6 months or CRS ≥2. In cases where both PFS and CRS data were available, PFS was prioritized as the primary indicator.

### 2.2 Patient selection and evaluation criteria

With approval from the Ethics Committee of Qingdao University Affiliated Hospital and after obtaining informed consent (QYFYWZLL29492), we conducted a retrospective study on patients with SOC who received platinum-based chemotherapy at our institution. The inclusion criteria were: a confirmed diagnosis of SOC, an Eastern Cooperative Oncology Group (ECOG) performance status of 0–2, completion of at least six cycles of platinum-based chemotherapy, and availability of comprehensive medical records. Clinical data were collected through medical chart reviews and patient follow-ups, and corresponding tumor tissue samples were obtained from the department of pathology. All treatments were administered in accordance with relevant clinical guidelines and drug protocols. Tumor staging prior to treatment was determined according to the 8th edition of the American Joint Committee on Cancer (AJCC) guidelines. Treatment responses were evaluated radiologically using the Response Evaluation Criteria in Solid Tumors (RECIST) version 1.1.

### 2.3 Single-cell RNA transcriptomics analysis

All single-cell RNA sequencing (scRNA-seq) analyses were performed in R (v4.2.0) with Seurat (v4.3.0) (Hao Y. et al., 2021). Cells with over 15% mitochondrial UMI content, fewer than 500 UMIs, or fewer than 200 detected genes were excluded. The gene expression matrix was normalized to total cellular UMI counts using the SCTransform method, followed by scaling, which corrects for technical variation due to differences in sequencing depth. Next, 3,000 highly variable features were selected for principal component analysis (PCA). To minimize batch effects, Harmony (v0.1.0) was used before PCA, ensuring that any technical variations were not confounding the biological analysis. The first 100 principal components were used for clustering at a resolution of 1, and the resulting clusters were visualized using t-SNE or UMAP. Cluster-specific markers were identified with the FindAllMarkers function using the Wilcoxon test, applying thresholds of log2-fold change >0.25 and min. pct >0.25. Cell types, including tumor cells, T cells, macrophages, and stromal cells, were annotated using SingleR (v1.4.1) and canonical marker genes from previous literature. Hallmark and KEGG pathway enrichment analyses were conducted using the GSVA (v1.50.5) (Hänzelmann et al., 2013) and msigdbr (v7.5.1) (Liberzon et al., 2015) R packages. The ClusterProfiler R package (v4.4.4) (Wu T. et al., 2021)was used for Gene Ontology (GO) enrichment analysis. SCENIC (v1.3.1) (Aibar et al., 2017) was employed for transcription factor analysis. Pseudotime trajectory analysis was carried out using the Monocle2 R package (v2.18.0) (Bray et al., 2016) and CytoTRACE (v0.3.3) (Gulati et al., 2020).

### 2.4 Combined analysis of single-cell RNA and bulk-RNA transcriptomics analysis

To integrate scRNA-seq data with bulk RNA-Seq data for ovarian cancer, bulk RNA-Seq data for tumor samples were obtained from TCGA, and normal ovarian bulk RNA-Seq data were sourced from GTEx. Sample annotations were standardized, and the expression matrix was filtered to retain genes expressed in at least 50% of samples with non-zero counts. Raw count data were normalized using the Trimmed Mean of M-values (TMM) method in the edgeR package to account for library size differences. To estimate cell-type proportions in bulk RNA-Seq data, cell-type-specific signatures derived from annotated scRNA-seq data were applied using CIBERSORT and ssGSEA algorithms. Specifically, CIBERSORT was employed to estimate the relative abundance of different cell types in the bulk RNA-seq data by comparing the gene expression patterns of these cell types with those in the bulk samples. For ssGSEA, gene sets corresponding to specific cell types were scored in bulk RNA-seq data to evaluate the enrichment of different cell types across tumor and normal samples. Differential expression analysis between tumor and normal samples was performed using the edgeR package, with thresholds set at FDR <0.05 and |log2FC| > 1 to identify significant differentially expressed genes (DEGs). Gene signatures derived from scRNA-seq analysis were scored in the bulk RNA-seq samples using the AddModuleScore function in Seurat. UMAP dimensionality reduction was performed to visualize tumor enrichment scores. Correlation analysis was conducted to examine gene expression relationships between different cell populations, and results were organized into a correlation matrix.

### 2.5 Combined analysis of single-cell RNA and spatial transcriptomics data analysis

Spatial transcriptomics data analysis was performed using R (v4.2.0), and the following packages were used: Seurat (v4.3.0), ggplot2 (v3.5.1), dplyr (v1.1.4), magrittr (v2.0.3), RColorBrewer (v1.1–3) packages (He et al., 2025; Liu et al., 2022). To normalize the ST data, we applied the SCTransform method for normalization, followed by integration steps involving selectIntegrationFeatures, prepSCTIntegration, findIntegrationAnchors, and integrateData to harmonize the datasets. The integration was performed to ensure compatibility between the different datasets. We then used an unsupervised clustering approach to identify spatially distinct spots. Cell population annotations were derived through deconvolution using scRNA-seq annotations to ensure consistency across the datasets, allowing for the alignment of cell identities between spatial and scRNA-seq data. Visualization of spatial expression patterns was accomplished using SpatialDimPlot and SpatialFeaturePlot functions, providing an in-depth view of cell distribution and activity across tissue sections. Additionally, for the spatial transcriptomics data, batch effects were controlled by integrating reference scRNA-seq data using the RCTD approach, which provides insights into the spatial distribution of cell types within the tumor microenvironment.

### 2.6 Cell-to-cell communication analysis

All analyses were performed in R (v4.2.0), and the following packages were used:

Seurat (v4.3.0), dplyr (v1.1.4), magrittr (v2.0.3), RColorBrewer (v1.1–3), tidyr (v1.3.1), patchwork (v1.2.0), and CellChat (v1.1.3) (He et al., 2025; Liu et al., 2022; Jin et al., 2021). To ensure robustness and minimize noise, ligand-receptor interactions expressed in fewer than 10 cells within specific cell groups were excluded. Interaction patterns were visualized using the netVisual function, generating bubble plots and signaling network diagrams. For pathways involving multiple ligand-receptor pairs, the netAnalysis contribution function was applied to evaluate the contribution of each pair. Gene expression levels of key ligands and receptors were displayed with PlotGeneExpression in violin plots to assess differential expression across cell groups. Non-negative Matrix Factorization (NMF) was applied within CellChat to extract major co-communication patterns, following the identification of significant signals using the identifyCommunicationPatterns function. Network centrality scores were calculated using netAnalysis_teCentrality to quantify the importance of cells within the communication network, and netAnalysis signalingRole network was used to identify dominant senders, receivers, mediators, and influencers in cell-cell communication networks.

### 2.7 Immunohistochemical staining analysis

Tumor tissues were fixed in 4% paraformaldehyde, dehydrated using an ethanol gradient (70%, 85%, 95%, 100%), embedded in paraffin, cut into 5 µm-thick sections using a paraffin-embedded tissue microtome (RM2235, Leica, Wetzlar, Germany), and dried in an oven at 60°C for 1 h. The sections were deparaffinized in xylene (2 changes, 10 min each), rehydrated through a descending ethanol gradient (100%, 95%, 85%, 70%; 5 min per step), and washed with phosphate-buffered saline (PBS, P3813-10 PAK, Sigma, USA). Antigen retrieval was performed using Tris-EDTA buffer (pH 9.0, 95°C, 20 min), followed by cooling in a water bath for 5 min.

Rabbit anti-STAR antibody (80751-1-RR, 1:500 dilution, Proteintech, Wuhan, China) was applied, and sections were incubated as per the manufacturer’s instructions. The percentage of STAR-positive cells was quantified using ImageJ software.

### 2.8 Statistical analysis

Data are presented as the mean ± SD. The Mann‒Whitney test or Student’s t-test was used to compare the differences between different groups. Correlation analyses employed the R function cor. test with Pearson’s method. Kaplan-Meier curves with log-rank tests were used for prognosis analysis. Differential expression in pseudotime or cell-type trajectories used negative binomial models with q-values <0.01 indicating significance. Data analysis and visualization were mainly performed using GraphPad Prism 9, R language, and R packages. The statistical significance was set at P < 0.05.

## 3 Results

### 3.1 Single-cell RNA sequencing reveals cellular composition of the tumor microenvironment

**FIGURE 1:**
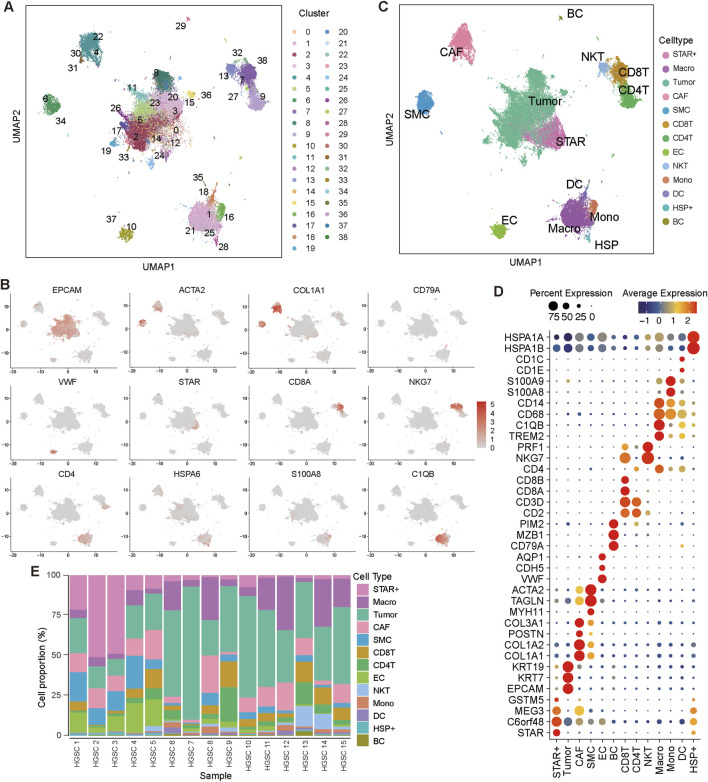
Identification of cell types in the SOC tumor microenvironment **(A)** Uniform Manifold Approximation and Projection (UMAP) visualization displaying all cell clusters. **(B)** UMAP visualization of cells colored by the expression levels of marker genes. **(C)** UMAP plot illustrating the main cell types identified in the tumor microenvironment (TME). **(D)** Dot plot depicting marker gene expression across the main cell types. **(E)** Proportions of the major cell types across all samples.

Fifteen SOC samples were collected and processed for scRNA-seq using standard protocols (Methods). After quality control, batch effect correction, and dimensionality reduction clustering, a total of 57,693 cells were retained for subsequent analyses and grouped into 39 distinct cell clusters (Figure 1A). These clusters were annotated into 13 major cell types based on canonical marker genes (Figures 1B, C). Specifically, the identified cell types were annotated based on canonical markers: epithelial cells (EPCAM, KRT19, KRT7), smooth muscle cells (MYH11, TAGLN, ACTA2), CAFs (COL1A1, COL1A2, POSTN), B cells (CD79A, MZB1), endothelial cells (VWF, CDH5), T cells (CD2, CD3D, CD8A, CD4), NK T cells (NKG7, PRF1), macrophages (CD68, C1QA, TREM2), monocytes (CD14, S100A8, S100A9), dendritic cells (CD1E, CD1C), and HSP + cells (HSPA6, HSP1B, HSPA1A).

Interestingly, we identified a unique cell population termed STAR + cells, which exhibited high expression of STAR, C6orf48, and MEG3, but lacked canonical epithelial markers (Figure 1D). These cells showed a close transcriptional relationship with tumor cells, suggesting a unique biological identity. Notably, STAR + cells were present in all SOC samples, as confirmed by their consistent detection across samples (Figure 1E). To further characterize the nature and functional relevance of STAR + cells, we performed an in-depth analysis.

### 3.2 STAR + cells as a special CAF subtype

**FIGURE 2:**
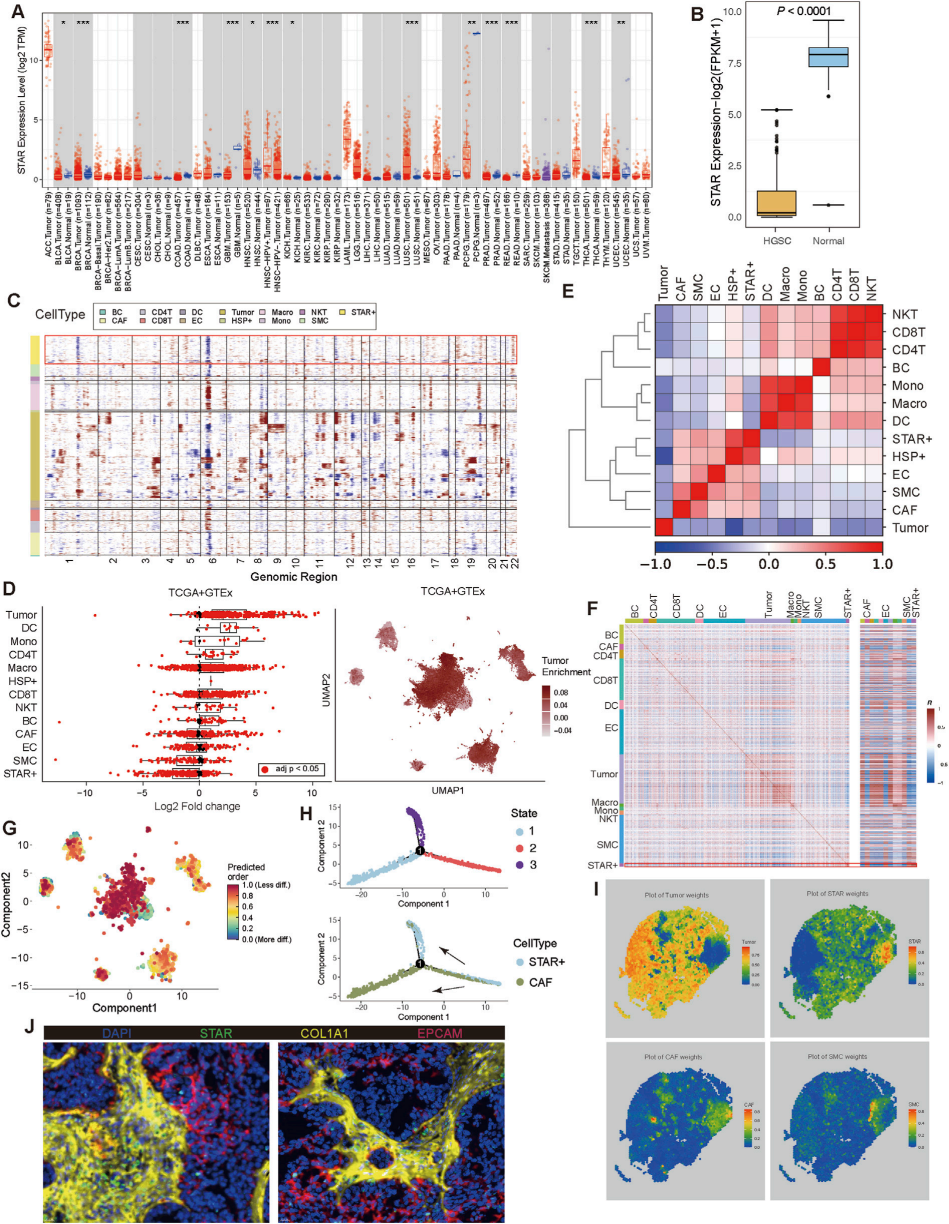
Identification and analysis of STAR + cells **(A)** The expression of STAR in pan cancers. **(B)** Expression level of STAR between SOC and normal samples. **(C)** Hierarchical heatmap from InferCNV analysis displaying large-scale copy number variations (CNVs) in different cells. **(D)** The enrichment of STAR + cell in tumor and normal ovarian sample. **(E, F)** Heatmap depicting the Pearson correlation coefficients among identified cell clusters in ScRNA-seq and bulk RNA-seq data. **(G)** Analysis of differentiation levels in major cell types. **(H)** Pseudotime trajectory analysis of STAR + cell and CAF. **(I)** Spatial transcriptomics reveals the spatial relationships among STAR + cell, tumor cell, CAF, and SMC. **(J)** Immunofluorescence staining of SOC showing the expression of STAR, COL1A1, and EPCAM. Blue represents DAPI, green represents STAR + cell, yellow represents COL1A1 (CAF), and red represents EPCAM (tumor cell).

A pan-cancer analysis revealed that STAR is expressed not only in ovarian cancer but also in other tumors, such as head and neck squamous cell carcinoma (HNSC), testicular germ cell tumors (TGCT), low-grade glioma (LGG), and lung squamous cell carcinoma (LUSC) (Figure 2A). In SOC, STAR expression was significantly lower than in normal ovarian tissues (Figure 2B). Previous studies have indicated that STAR is primarily expressed in ovarian granulosa and theca cells. To further explore the characteristics of STAR + cells, we analyzed an independent dataset containing SOC and normal tissues. This analysis showed that although STAR + cells share some transcriptional similarity with granulosa/theca cells, they are a distinct cell type more closely related to fibroblasts (Supplementary Figures S1A, B).

Further examination revealed that while STAR expression is high in both STAR + cells and granulosa/theca cells, their marker gene expression profiles differ. STAR + cells exhibited elevated expression of fibroblast-associated genes such as COL3A1 and COL1A1, while granulosa/theca cells predominantly expressed GJA1, LHCGR, and LSD3B2 (Supplementary Figures S1C, D). This expression pattern is consistent with the STAR + cell profile observed in this study (Supplementary Figure S1E). Single-cell sequencing further confirmed that STAR expression in STAR + cells and granulosa/theca cells was significantly lower in SOC tissues compared to normal tissues (Supplementary Figure S1F). These findings indicate that the STAR + cells identified in this study are distinct from granulosa and theca cells.

To distinguish STAR + cells from tumor cells, we performed inferCNV analysis, which showed that STAR + cells exhibited low CNV levels, similar to CAFs and smooth muscle cells (SMCs), whereas tumor cells displayed high CNV levels indicative of malignancy (Figure 2C). Additionally, deconvolution of scRNA-seq annotations into TCGA and GTEX bulk RNA-seq data revealed that STAR + cells are predominantly present in normal tissues, with significantly lower enrichment in tumor tissues compared to tumor cells (Figure 2D). These results support that STAR + cells are non-malignant, despite their transcriptional and spatial proximity to tumor cells.

Correlation analyses using both scRNA-seq and bulk RNA-seq data demonstrated that STAR + cells are closely associated with CAFs, SMCs, and endothelial cells (Figures 2E, F). Pseudotime trajectory analysis showed that STAR + cells are more differentiated compared to tumor cells, suggesting a higher degree of maturity. STAR + cells shared partial developmental trajectories with CAFs but exhibited distinct developmental endpoints (Figures 2G, H, Supplementary Figure S1G). Spatial transcriptomics suggest that STAR + cells are spatially proximal to CAFs, but they showed stronger spatial association with tumor cells compared to CAFs (Figure 2I). To further validate this, IF was conducted, which confirmed that STAR is predominantly expressed in CAFs, with a small amount of expression observed in tumor cells (Figure 2J).

In summary, these findings suggest that STAR + cells represent a distinct CAF subtype and are closely associated with tumor cells.

### 3.3 STAR + cells are associated with platinum-based chemotherapy sensitivity in SOC

**FIGURE 3:**
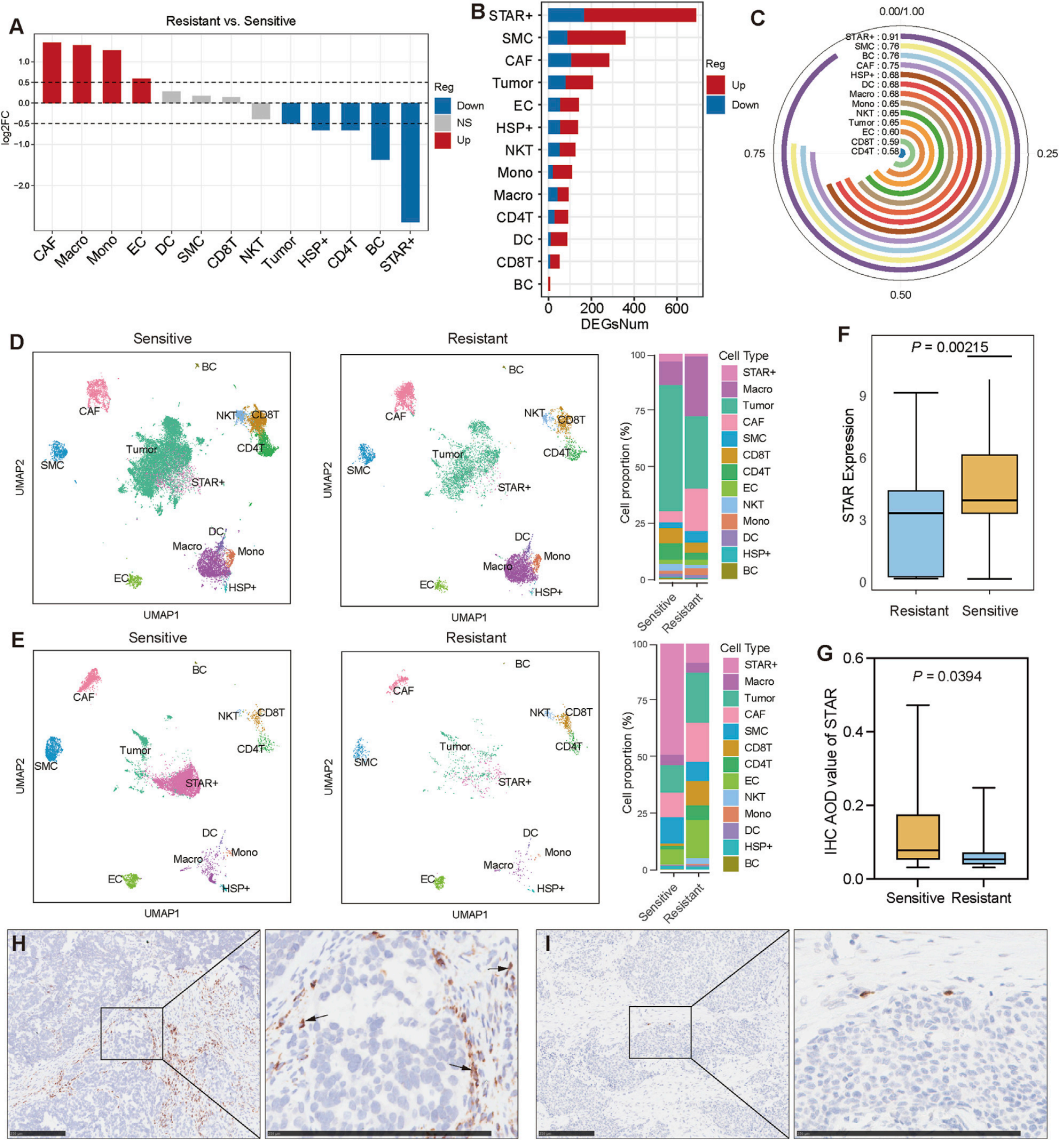
STAR + cells are associated with platinum-based chemotherapy sensitivity in SOC **(A)** Differential abundance of cell types between chemotherapy-resistant and chemotherapy-sensitive SOC patients. **(B)** Number of differentially expressed genes (DEGs) in each cell type between chemotherapy-resistant and chemotherapy-sensitive patients. **(C)** AUC-based evaluation of cell-type specificity in distinguishing chemotherapy-resistant from chemotherapy-sensitive patients. **(D, E)** UMAP visualization and cell-type composition showing the differences in cell proportions between chemotherapy-sensitive and chemotherapy-resistant SOC patients before and after treatment. **(F)** Comparison of STAR expression levels between chemotherapy-sensitive and chemotherapy-resistant patients using bulk RNA-seq data. **(G)** Comparison of STAR AOD values between chemotherapy-sensitive and chemotherapy-resistant patients based on IHC data. **(H, I)** Representative IHC image of STAR in chemotherapy-sensitive and chemotherapy-resistant patient.

To investigate the differences in the TME between chemotherapy-sensitive and chemotherapy-resistant SOC groups, we conducted a detailed analysis. The results showed that STAR + cells were the most significantly different cell population between the two groups, with a higher proportion of STAR + cells in the chemotherapy-sensitive group compared to the resistant group (Figure 3A). Differential gene expression analysis further revealed that STAR + cells exhibited the highest number of differentially expressed genes (DEGs) between the two groups, with these DEGs demonstrating high specificity and a strong ability to distinguish chemotherapy-sensitive from resistant groups (Figures 3B, C).

To account for potential variations due to sampling time points, we separately compared samples collected before and after chemotherapy. In pre-chemotherapy samples, the proportions of tumor cells, STAR + cells, and T lymphocytes were higher in the sensitive group, while CAFs and macrophages were more abundant in the resistant group (Figure 3D). In post-chemotherapy samples, tumor cell proportions were significantly reduced in the sensitive group, accompanied by decreases in CD4 T cells, CD8 T cells, and NKT cells. However, the proportion of STAR + cells remained higher in the sensitive group compared to the resistant group (Figure 3E).

Independent bulk RNA-seq data also showed that STAR expression levels were higher in the chemotherapy-sensitive group compared to the resistant group (Figure 3F). Moreover, analysis of tumor specimens from SOC patients receiving platinum-based chemotherapy at our center confirmed that STAR expression was significantly higher in the sensitive group (N = 24) compared to the resistant group (N = 20) (Figures 3G–I).

These findings suggest that STAR + cells are positively associated with platinum-based chemotherapy sensitivity in SOC and may enhance the cytotoxic effects of chemotherapy on tumor cells.

### 3.4 STAR + cells are positively associated with chemotherapy response and prognosis in SOC

Given the close association between STAR + cells and platinum-based chemotherapy sensitivity in SOC, we further hypothesized that STAR + cells may influence clinical outcomes in patients.

**FIGURE 4:**
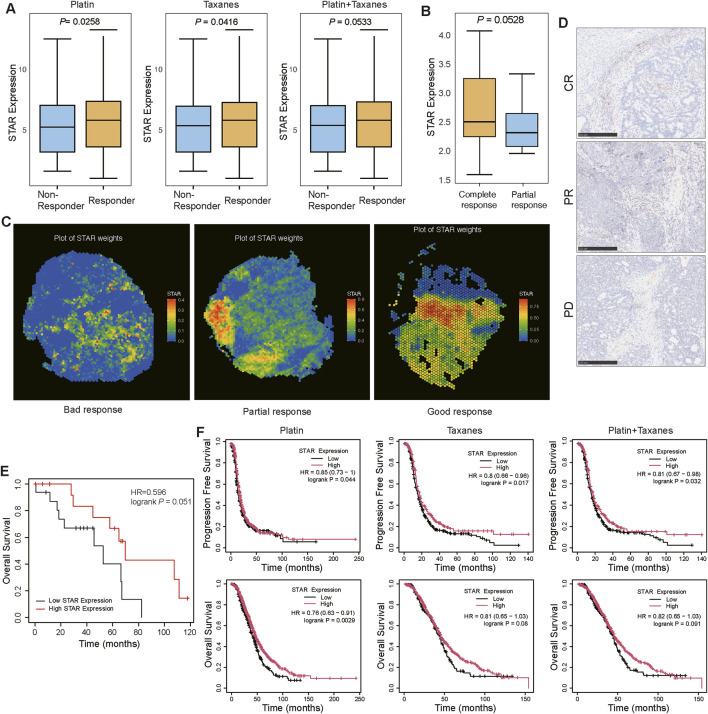
STAR + cells are positively associated with prognosis in SOC **(A)** Comparison of STAR expression levels between responders and non-responders to platinum, taxane, and platinum-taxane combination chemotherapy at 12 months post-treatment. **(B)** Comparison of STAR expression levels between patients with complete response (CR) and partial response (PR) to platinum-based chemotherapy. **(C)** Spatial distribution of STAR weights in SOC patients showing bad, partial, and good chemotherapy responses. **(D)** Representative IHC images of STAR expression in SOC patients achieving complete response (CR), partial response (PR), and progressive disease (PD) following platinum-based chemotherapy. **(E)** Kaplan-Meier plot showing the association between STAR expression levels and overall survival in ovarian cancer patients. **(F)** Kaplan-Meier plot showing the association between STAR expression levels and progression-free survival and overall survival in SOC patients receiving platinum, taxane, and platinum-taxane combination chemotherapy.

To validate this hypothesis, we analyzed transcriptomic data from patients who received platinum, taxane, or platinum-taxane combination chemotherapy at 12 months post-treatment. The results showed that STAR expression levels were higher in patients who responded well to treatments compared to non-responders (Figure 4A). Moreover, among patients treated with platinum-based chemotherapy, those achieving complete response (CR) exhibited significantly higher STAR expression levels compared to patients with partial response (PR) (Figure 4B). Spatial transcriptomic analysis revealed that the proportion of STAR + cells gradually increased in patients with poor response, partial response, and good response to chemotherapy (Figure 4C). Similarly, a comparable trend was observed in post-chemotherapy patients achieving progressive disease (PD), PR, or CR (Figure 4D). These results indicate that STAR + cells are positively associated with the response to platinum-based chemotherapy in SOC, with higher STAR expression levels potentially corresponding to improved chemotherapy efficacy.

Further analysis of the relationship between STAR expression and the clinical prognosis of SOC patients revealed that, in the overall ovarian cancer cohort, patients with high STAR expression had significantly longer overall survival (OS) compared to those with low STAR expression (Figure 4E). Subgroup analyses based on platinum, taxane, or platinum-taxane combination chemotherapy consistently showed that high STAR expression is a protective factor for SOC patients, correlating with better progression-free survival (PFS) and OS (Figure 4F).

In conclusion, STAR + cells are positively associated with platinum-based chemotherapy sensitivity and clinical prognosis in SOC, suggesting that STAR + cells may serve as potential biomarker for predicting chemotherapy response and prognosis in SOC patients.

### 3.5 Platinum-based chemotherapy increases STAR + cell levels

We have demonstrated that STAR + cells are positively associated with chemotherapy efficacy and patient prognosis. Based on these findings, we hypothesized that platinum-based chemotherapy may influence the levels of STAR + cells.

**FIGURE 5:**
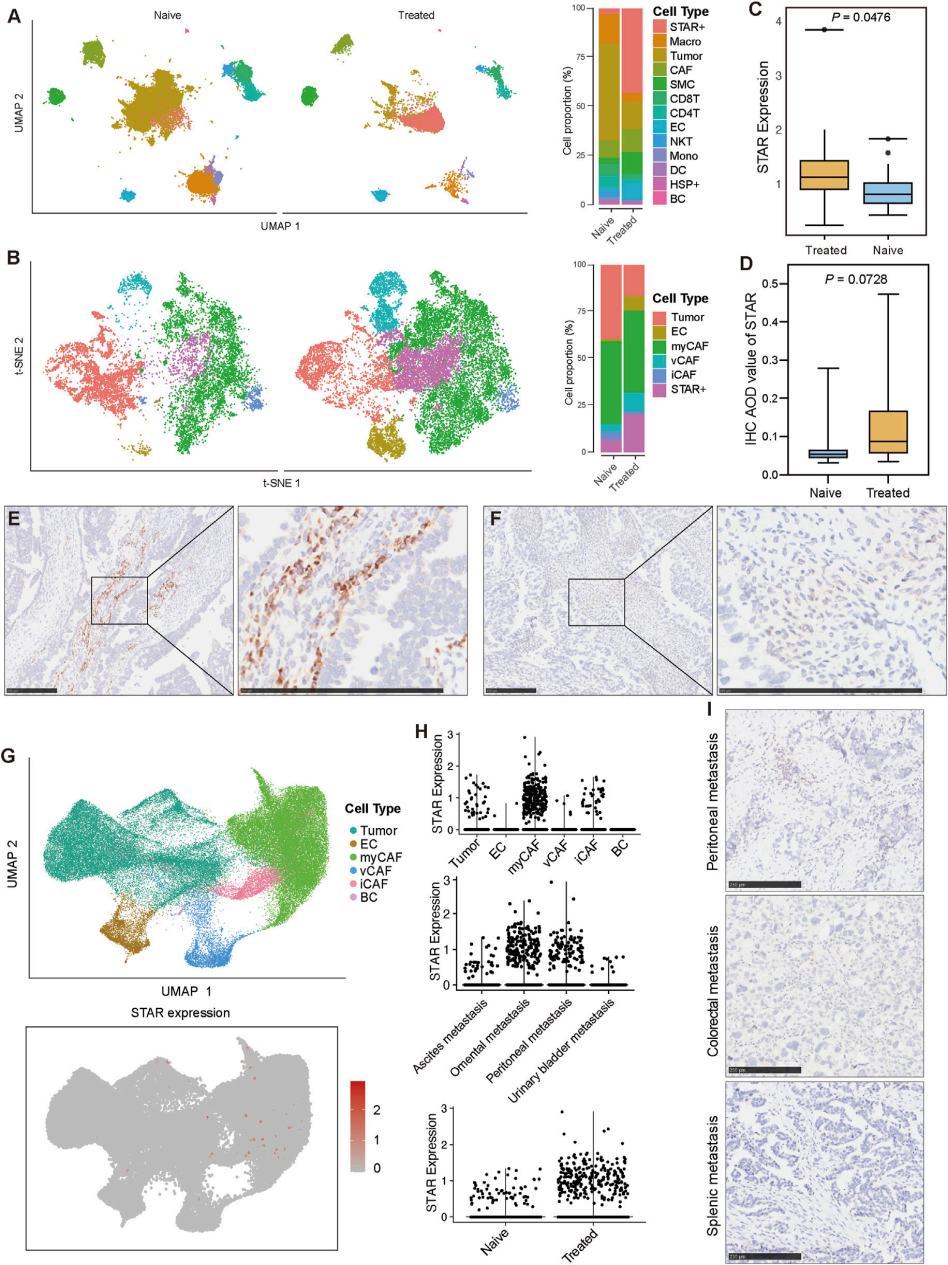
Variation of STAR + cell levels in chemotherapy-naïve and chemotherapy-treated primary and metastasis SOC samples **(A)** UMAP visualization and cell-type composition illustrating the differences in cell proportions between pre-chemotherapy and post-chemotherapy SOC patients. **(B)** UMAP visualization and cell-type composition showing the differences in cell proportions between paired pre-chemotherapy and post-chemotherapy SOC patients. **(C)** Comparison of STAR expression levels between pre-chemotherapy and post-chemotherapy SOC patients using bulk RNA-seq data. **(D)** Comparison of STAR AOD values in patients based on IHC data. **(E, F)** Representative IHC image of STAR in pre-chemotherapy and post-chemotherapy SOC patient. **(G)** UMAP visualization the expression of STAR in metastasis SOC samples. **(H)** Violin plots showing STAR expression levels across different cell types (top), metastatic tumor locations (middle), and pre-chemotherapy and post-chemotherapy metastatic SOC samples (bottom). **(I)** IHC image of STAR in different metastatic tumor locations.

To test this hypothesis, patients were divided into two groups: pre-chemotherapy (naïve group) and post-chemotherapy (treated group), based on sampling time points. The results showed a significant reduction in tumor cells following chemotherapy, confirming the anti-tumor effect of platinum-based treatment. In contrast, the proportion of STAR + cells increased significantly after chemotherapy (Figure 5A). Considering that the pre-chemotherapy and post-chemotherapy samples in this dataset were not paired, we validated these findings using another dataset containing matched pre-chemotherapy and post-chemotherapy primary tumor samples from the same patients. This analysis also revealed a decrease in tumor cells and a significant increase in STAR + cells after chemotherapy (Figure 5B). Independent bulk RNA-seq data further confirmed that STAR expression levels were significantly elevated in post-chemotherapy samples compared to pre-chemotherapy (Figure 5C). Analysis of IHC data from patients at our center similarly validated these findings, showing increased STAR expression in post-chemotherapy (N = 23) compared to pre-chemotherapy (N = 21) (Figures 5D–F).

Furthermore, to investigate the behavior of STAR + cells in metastatic SOC lesions, we analyzed metastatic tumor samples. The results showed that STAR expression levels were generally low in metastases, and no distinct STAR + cell population was identified (Figure 5G). Further analysis revealed that STAR was primarily expressed in myCAFs, with varying levels across metastatic sites. Specifically, STAR expression was higher in omental and peritoneal metastatic lesions (Figure 5H). IHC analysis further confirmed the low expression of STAR in metastatic tumors (Figure 5I). However, despite the overall low levels, STAR expression in metastatic tumors was still higher in post-chemotherapy samples compared to pre-chemotherapy samples (Figure 5H).

In conclusion, these results further confirm the close relationship between STAR + cells and platinum-based chemotherapy, suggesting that the clinical benefit of platinum-based treatment in SOC patients may be associated with an increase in STAR + cells.

### 3.6 STAR + cells influence drug efficacy through metabolic pathways

**FIGURE 6:**
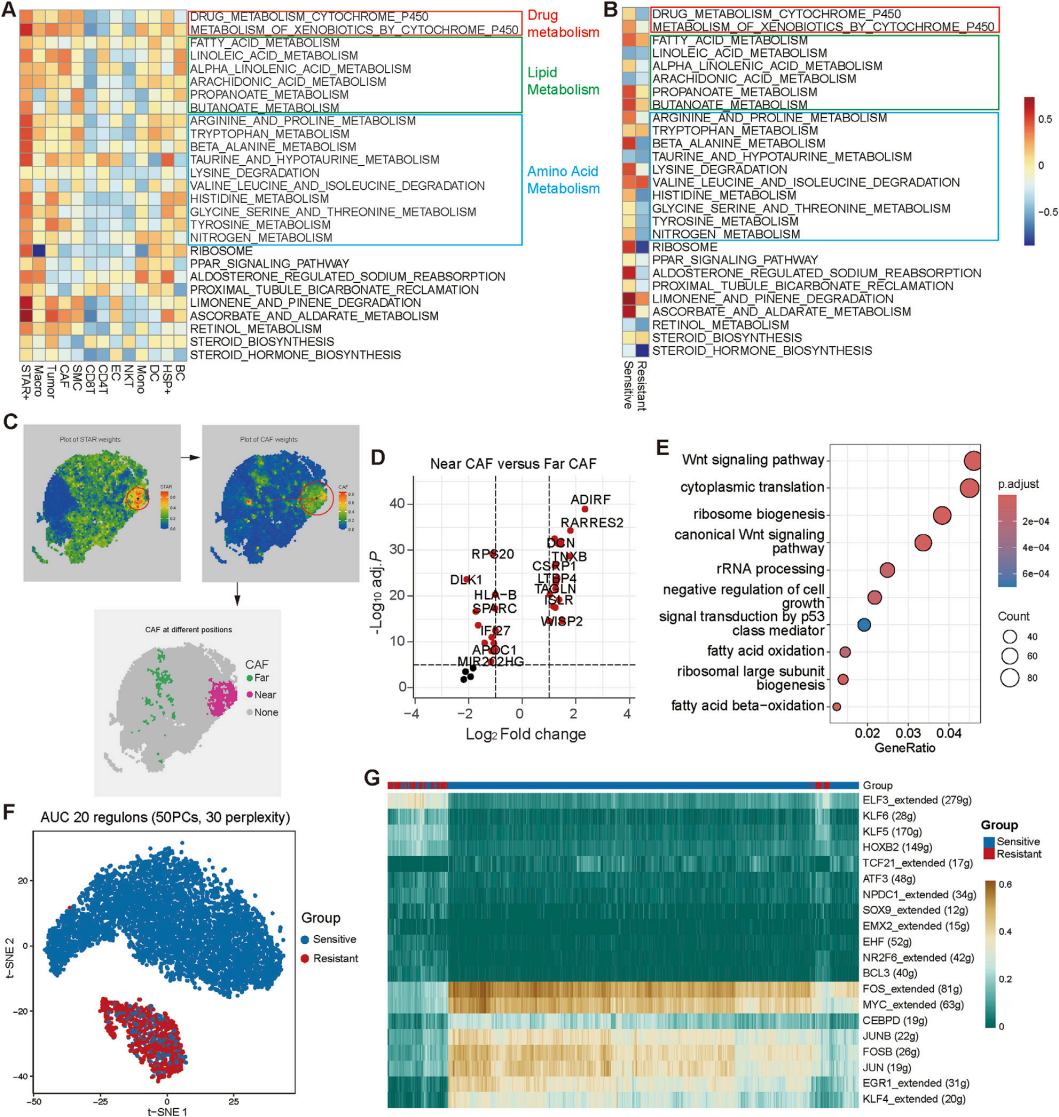
STAR + cells influence chemotherapy response through metabolic pathways and transcription factor activation. **(A)** Heatmaps showing pathways enriched in cell types. **(B)** Heatmaps showing pathways enriched of STAR + cells in chemotherapy-sensitive and chemotherapy-resistant patients. **(C)** Spatial distribution of STAR + cells and their spatial proximity to CAFs in the SOC TME. **(D)** Volcano plot showing differentially expressed genes (DEGs) between near CAFs and far CAFs. **(E)** Bubble plot showing GO enrichment analysis of STAR + cells. **(F, G)** Transcription factor analysis of STAR + cellsin chemotherapy-sensitive and chemotherapy-resistant groups.

To investigate how STAR + cells affect the efficacy of platinum-based chemotherapy in SOC, we conducted a comprehensive functional analysis of STAR + cells. GSVA analysis revealed that, compared to other cell types, STAR + cells were significantly enriched in metabolic pathways, including P450 metabolism, lipid metabolism, and amino acid metabolism (Figure 6A). Further analysis showed that, in the chemotherapy-sensitive group, STAR + cells exhibited higher enrichment in these pathways compared to the resistant group (Figure 6B). These findings suggest that STAR + cells may regulate drug metabolism, energy supply, and oxidative stress responses, thereby influencing chemotherapy drug efficacy (Gougis et al., 2021).

The correlation between STAR + cells and metabolic pathways was further validated by spatial transcriptomic data. Previous spatial transcriptomic analysis revealed the spatial proximity of STAR + cells to CAFs; however, CAFs exhibited spatial heterogeneity. To address this, we classified CAFs into Far CAFs and Near CAFs based on their spatial distance from STAR + cells and performed differential gene expression analysis (Figure 6C). The results showed that Near CAFs exhibited significantly higher expression of metabolism-related genes such as ADIRF and PARRES2 (Figure 6D).

GO enrichment analysis indicated that, in addition to fatty acid metabolism pathways, STAR + cells were also involved in Wnt signaling pathways and p53 signal transduction, which are associated with tumor progression (Figure 6E). Moreover, we observed potential functional differences in STAR + cells between the chemotherapy-sensitive and resistant groups. For example, STAR + cells in the sensitive group displayed higher transcription factor activity (Figure 6F). Specifically, the transcription factors FOS/FOSB, JUN/JUNB, and EGR1, which are closely linked to the p53 signaling pathway, exhibited significantly elevated activity in the sensitive group (Figure 6G).

In conclusion, STAR + cells may influence chemotherapy efficacy by modulating metabolism-related pathways in the TME, as well as key signaling pathways such as Wnt and p53.

### 3.7 STAR + cells promote chemotherapy efficacy by suppressing wnt signaling

**FIGURE 7:**
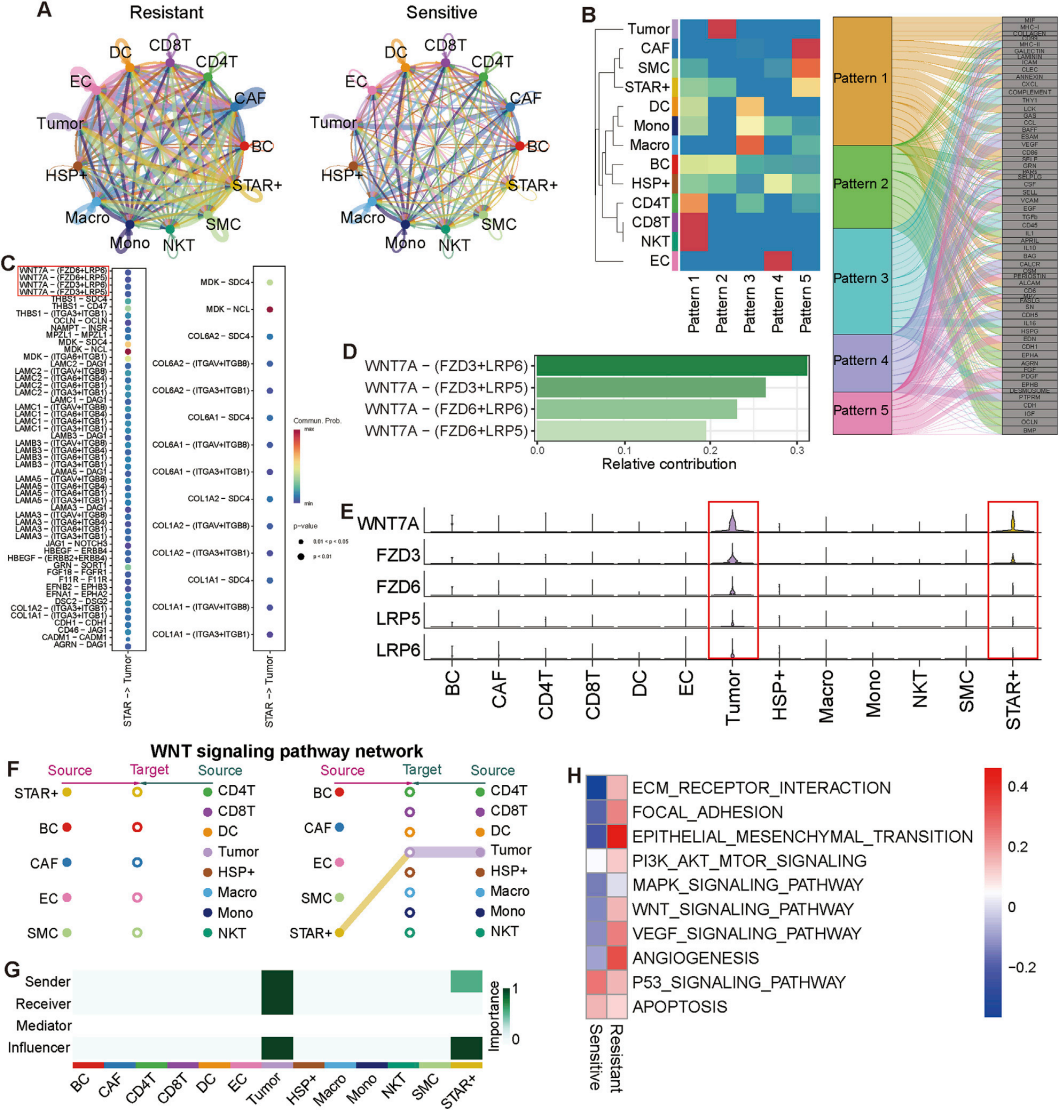
Cell-cell communication between tumor cell and STAR + cells **(A)** Cell-cell communication networks showing differences between chemotherapy-sensitive and chemotherapy-resistant groups. **(B)** Communication patterns of cell types. **(C)** Dot plot showing the main significant ligand-receptor (L-R) pairs between tumor cells and STAR + cells. The dot color and size represent the calculated communication probability and p-values. p-values are computed from a one-sided permutation test. **(D)** The L-R paires Wnt signaling include, and the relative importance of L-R paires in Wnt signaling. **(E)** WNT-related gene expression levels in cell types. **(F)** Hierarchical plot shows the inferred intercellular communication network for Wnt signaling. This kind of plot consists of two parts: The left side focusing on signaling targeting stromal cells and B cells while the right side focusing on signaling targeting tumor cells and immune cells. Solid and open circles represent source and target, respectively. Circle sizes are proportional to the number of cells in each cell group and edge width represents the communication probability. Edge colors are consistent with the signaling source. **(G)** Heatmap shows the relative importance of different cell-types based on the computed four network centrality measures of Wnt signaling network. **(H)** Heatmap showing enriched pathways in chemotherapy-sensitive and chemotherapy-resistant tumor cells.

We further analyzed the cell-cell communication between STAR + cells and other cell types within the TME. The chemotherapy-sensitive group showed significantly fewer cell-cell communications than the resistant group, indicating that improved chemotherapy efficacy reduced the complexity of cellular interactions. Despite a higher proportion of STAR + cells in the sensitive group, their communication numbers also decreased (Figure 7A). STAR + cells shared similar communication patterns with CAFs and SMCs, primarily enriched in Pattern 5 (Figure 7B), confirming their close relationship with CAFs.

We observed significant differences in ligand-receptor pairs (LRPs) between STAR + cells and tumor cells in the sensitive and resistant groups. In the resistant group, the primary LRPs included WNT7A-FZD6 +LRP6 and LAMA/B/C-ITGAC (Figure 7C), which were absent in the sensitive group, replaced by LRPs associated with p53 signaling, such as COL1A1-ITGA3 +ITGB1 (Figure 7C). WNT7A-FZD6/FZD3+LRP6/LRP5, critical components of the canonical Wnt signaling pathway, were exclusively expressed in STAR + cells and tumor cells (Figures 7D, E). The Wnt-FZD + LRP LRPs play an important role in the Wnt signaling pathway. Specifically, Wnt signaling activates signal transduction by binding with the FZD receptor and the LRP5/6 co-receptor, leading to the dimerization or aggregation of these two receptors. This mechanism is crucial for intercellular communication, especially in the TME, where Wnt signaling regulates the interaction between tumor cells and CAFs (Nusse and Clevers, 2017; Ren et al., 2021). Moreover, it has also been observed in the nervous system that Wnt7a activates β-catenin signaling through binding with FZD + LRP6/LRP5 (Zhao et al., 2015).

Additionally, we found that Wnt signaling transmitted unidirectionally from STAR + cells to tumor cells via paracrine signaling. Tumor cells also regulated their own Wnt signaling through autocrine signaling (Figures 7F, G). Pathway analysis of tumor cells showed that, in the resistant group, Wnt signaling, angiogenesis, and invasion-related pathways were upregulated, while in the sensitive group, p53 signaling and apoptosis pathways were elevated (Figure 7H).

In conclusion, these results support our hypothesis that STAR + cells influence tumor behavior by regulating Wnt and p53 signaling, enhancing the anti-tumor effects of platinum-based chemotherapy.

## 4 Discussion

Platinum-based chemotherapy remains the cornerstone of treatment for SOC, yet chemoresistance remains a significant barrier to improving patient outcomes, as most patients eventually relapse with platinum-resistant disease (Richardson et al., 2023). This study presents several key advancements in understanding SOC chemoresistance by identifying STAR + cells as a novel CAF subtype and conducting a comprehensive analysis of their characteristics, functions, and underlying mechanisms. Notably, this is the first study to establish the relationship between STAR + cells and platinum-based chemotherapy sensitivity, offering mechanistic insights into their potential roles within the TME.

STAR is a mitochondrial protein primarily involved in steroidogenesis, facilitating the transport of cholesterol into mitochondria for steroid hormone synthesis (Clark et al., 1994). Under normal physiological conditions, STAR is highly expressed in ovarian granulosa and luteal cells, where its expression is tightly regulated during the luteal phase, playing a key role in progesterone production during luteal development and regression (Dou et al., 2016). STAR is also associated with hormone signaling pathways involving NR4A1, CEBPD, and ADAMTS4 (Jones et al., 2024). Recent studies have identified STAR + cells, defined as theca and stroma (T&S) cells that co-express DCN and STAR, as linked to RNA and protein synthesis processes, contributing to ovarian aging (Wu M. et al., 2024). Despite these findings, the role of STAR in ovarian cancer, particularly in SOC, remains largely unexplored. With the advancement of single-cell sequencing technology, Qian Hao et al. first reported the expression of STAR in fibroblasts of SOC (Hao Q. et al., 2021), and Nele Loret et al. further described STAR + cells as a unique CAF subtype in SOC (Loret et al., 2022). However, the functional significance and detailed characteristics of STAR + cells remain to be fully elucidated.

Our findings demonstrate that STAR + cells are distinct from both stromal and tumor cell populations. While STAR has traditionally been associated with granulosa and luteal cells, our analysis reveals that STAR + cells possess a unique transcriptional profile, differentiating them from granulosa/luteal lineages. Furthermore, both spatial and transcriptional analyses and IF analysis of SOC patient samples confirmed that STAR + cells are a unique CAF subtype, and that STAR + cells are in closer proximity to tumor cells than typical CAFs, suggesting that they may perform distinct and specialized functions within the TME, setting them apart from traditional CAF populations.

We found that STAR + cells were enriched in chemotherapy-sensitive patients compared to resistant ones, and this was validated using scRNA-seq, bulk RNA-seq, and IHC. The differential gene expression in STAR + cells between the two groups was highly specific, suggesting their role in modulating chemotherapy responses. These findings support STAR + cells as a potential biomarker for chemotherapy sensitivity. Although STAR + CAFs have only recently been described, no prior studies have focused on their relationship with chemotherapy responses. Previous research showed that chemotherapy reduced STAR expression in granulosa cells in normal ovaries (El Andaloussi et al., 2018), and in esophageal cancer, elevated STAR expression was linked to chemotherapy resistance (Holloway et al., 2024). However, these studies did not investigate STAR + CAFs specifically. Our study provides new insights into the role of STAR + cells in chemotherapy sensitivity in SOC.

STAR + cells influence chemotherapy sensitivity by modulating key metabolic pathways. GSVA analysis revealed significant enrichment of STAR + cells in P450 metabolism, lipid metabolism, and amino acid metabolism pathways, which regulate drug metabolism, energy supply, and oxidative stress responses (Klomp et al., 2020; Snaebjornsson et al., 2020; Ling et al., 2023). Notably, STAR + cells in chemotherapy-sensitive patients exhibited higher enrichment in these pathways compared to resistant patients, suggesting that STAR + cells may enhance chemotherapy efficacy by regulating metabolic processes that increase tumor vulnerability to platinum-based drugs. Additionally, cell-cell communication analysis revealed that in chemotherapy-resistant patients, STAR + cells interact with tumor cells through Wnt signaling, specifically via ligand-receptor pairs such as WNT7A-FZD6/FZD3-LRP6/LRP5, components of the canonical Wnt pathway. Wnt signaling promotes tumor proliferation, angiogenesis, and chemoresistance (Parsons et al., 2021; Wu J. et al., 2024; Liu et al., 2021). However, in chemotherapy-sensitive patients, Wnt signaling was suppressed, indicating that STAR + cells may enhance chemotherapy efficacy by inhibiting Wnt signaling in tumor cells.

This relationship among STAR + cells, Wnt signaling, and tumor cells has also been reported in other studies. For example, in colorectal cancer, CAFs secrete exosomes that activate the Wnt signaling pathway in tumor cells, contributing to chemoresistance (Hu et al., 2015; Hu et al., 2019), although the specific CAF subtypes responsible for this effect remain unclear. In bladder cancer, Zikun Ma et al. identified SLC14A1+ CAFs through single-cell sequencing, which impart stemness to tumor cells via the Wnt pathway, thus enhancing chemotherapy resistance (Ma et al., 2022).

A key finding was the increase in STAR + cell proportions following platinum-based chemotherapy. While chemotherapy reduced tumor cell numbers, STAR + cells became more prevalent in both primary tumors and bulk RNA-seq analyses. This paradoxical enrichment may reflect STAR + cells’ role in creating a microenvironment less favorable for tumor survival during chemotherapy. However, STAR expression was lower in metastatic tumors, suggesting spatial or functional heterogeneity depending on the tumor context. The interaction between STAR + cells and tumor cells highlight the complexity of the TME in SOC and underscores the need to identify specific CAF subtypes that impact treatment outcomes. Previous studies have shown CAF functional heterogeneity in matrix remodeling, immune regulation, and drug resistance (Wu F. et al., 2021; Caligiuri and Tuveson, 2023).

Although this study provides a comprehensive exploration of STAR + cells as an exploratory study, there are several limitations and challenges to consider. First, technical difficulties in isolating and culturing STAR + cells, coupled with the lack of established cell lines, hinder short-term functional validation. We plan to conduct further functional studies, including co-culture experiments and interference assays, once STAR + cells are successfully isolated and stabilized. Additionally, we aim to validate the role of STAR + cells in chemotherapy efficacy through metabolomics analysis and by investigating their interaction with key molecules in the Wnt and p53 pathways using techniques like qPCR or Western blot. We also intend to use patient-derived xenograft models to further explore STAR + cells’ role in chemotherapy sensitivity and their potential as therapeutic targets for SOC. Secondly, despite employing effective multi-omics integration approaches, our analysis has inherent limitations, such as reliance on algorithms like CIBERSORT and ssGSEA, which may not fully capture rare or poorly annotated cell types. Thirdly, the small clinical sample size and focus on SOC limit our ability to assess the correlation between STAR + cells and clinical features, and further exploration of STAR + cells in other tumor types is needed, with ongoing sample collection and collaboration to validate these findings. Lastly, while STAR + cells show potential as a chemotherapy efficacy biomarker in SOC, challenges such as the development of reliable protocols for isolation, validation in larger multi-center trials, and cost-effective detection methods must be addressed before their practical application.

In conclusion, this study provides novel insights into the biology and clinical significance of STAR + cells in SOC. We demonstrate, for the first time, that STAR + cells are positively associated with platinum-based chemotherapy sensitivity and may exert their effects through metabolic modulation and suppression of Wnt signaling, suggesting their potential as a biomarker for predicting chemotherapy efficacy in SOC.

## Data Availability

The datasets presented in this study can be found in online repositories. The names of the repository/repositories and accession number(s) can be found in the article/Supplementary Material.
