# Supplementary material for: Multi-omics analysis of the dynamic role of STAR+ cells in regulating platinum-based chemotherapy responses and tumor microenvironment in serous ovarian carcinoma
Source: Front Pharmacol. 2025 Mar 3;16:1545762. doi: 10.3389/fphar.2025.1545762 (PMC11911460; doi:10.3389/fphar.2025.1545762)
Supplement: Supplementary file 1 [file Image1.pdf]

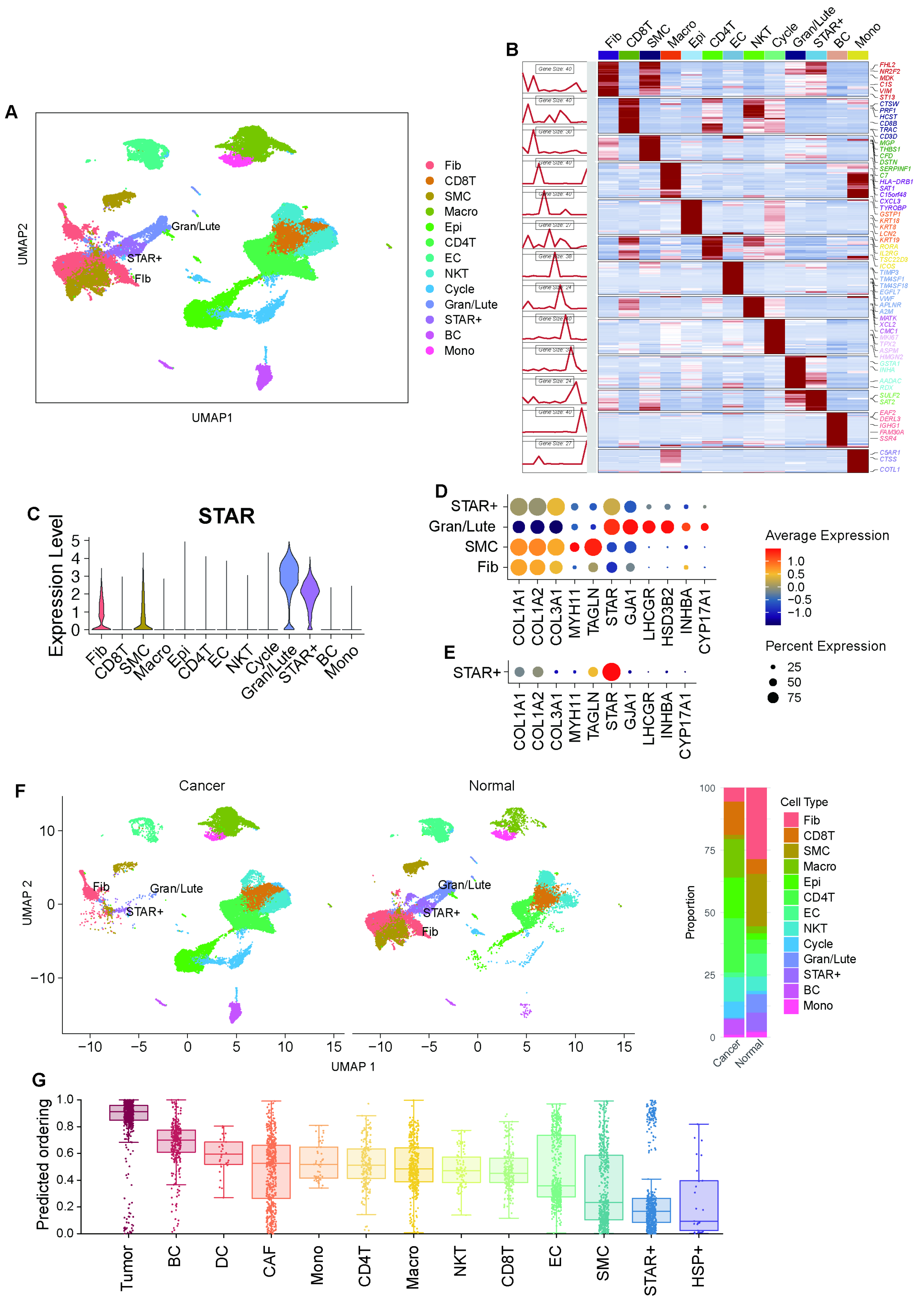

Figure S1. Identification and annotation of cell types in SOC and normal samples

(A) UMAP visualization of cell type in SOC and normal ovarian samples. (B) Heatmap displaying the heterogeneity and expression of genes across cell types. (C) Violin plots illustrating the expression levels STAR across different cell types. (D) Dot plot showing the gene expression profile of STAR+ cells and granulosa/theca cells in SOC and normal ovarian samples. (E) Dot plot showing the gene expression profile of STAR+ cells in SOC samples. (F) UMAP visualization and cell-

type composition showing the differences in cell proportions between SOC and normal samples. (G) Analysis of differentiation levels in major cell types.
